# Rasch analysis of the brief Michigan Hand Questionnaire in patients with thumb osteoarthritis

**DOI:** 10.1186/s12891-022-05478-4

**Published:** 2022-06-08

**Authors:** Shannon C. Killip, Joy C. MacDermid, Robbert M. Wouters, Kathryn E. Sinden, Rebecca E. Gewurtz, Ruud W. Selles, Tara L. Packham

**Affiliations:** 1 School of Rehabilitation Science, McMaster University, 1400 Main Street West IAHS 403, Hamilton, ON L8S 4L8 Canada; 2 Physical Therapy and Surgery, Western University, London, ON Canada; 3 Clinical Research Lab, Hand and Upper Limb Centre, St. Joseph’s Health Centre, London, ON Canada; 4 Departments of Rehabilitation Medicine and Plastic, Reconstructive and Hand Surgery, Erasmus MC Rotterdam, Dr. Molewaterplein 40, 3015 GD Rotterdam, The Netherlands; 5 Center for Hand Therapy, Handtherapie Nederland, Eindhoven, The Netherlands; 6 School of Kinesiology, Lakehead University, 955 Oliver Rd, Thunder Bay, Thunder Bay, ON P7B 5E1 Canada

**Keywords:** Brief Michigan Hand Questionnaire, Rasch analysis, Osteoarthritis, Psychometrics, Reliability and validity, Hand injuries, Patient-reported outcome measures

## Abstract

**Background:**

The brief Michigan Hand Questionnaire (brief MHQ) is a 12-item self-reported measure of hand function for patients with hand disorders which has been validated using Classical Test Theory. Rasch analysis can provide more detailed psychometric information. The purpose of this Rasch analysis is to assess the psychometric properties of the brief MHQ for patients with thumb osteoarthritis, and to make recommendations for improvements to the questionnaire if needed.

**Methods:**

The Michigan Hand Questionnaire and demographic data were collected from 923 thumb osteoarthritis patients treated in specialized clinics for hand surgery and therapy in the Netherlands. Rasch analysis was performed on the 12 items of the brief MHQ using RUMM 2030 to assess the fit of the brief MHQ to the Rasch model. To determine fit, analysis of fit summary statistics, individual person fit and individual item fit were assessed. Threshold distributions were assessed to identify if any items required rescoring. The Person Separation Index was calculated to measure reliability of the questionnaire. Differential item functioning was assessed to identify item bias, and Principal Component Analysis was performed to identify unidimensionality and local dependence.

**Results:**

The brief MHQ showed misfit (χ^2^ = 1312.5, *p* < 0.0001) with 6 items having disordered thresholds and 9 items requiring rescoring. After deleting 3 of the rescored items due to significant item fit residuals, the brief MHQ had an acceptable reliability (Cronbach’s alpha = 0.79). Misfit to the model (χ^2^ = 49.6, *p* = 0.0001), multidimensionality (10.2% of t-tests were significant), and item bias from non-uniform differential item functioning for 7 items across many person variables were still found.

**Conclusion:**

Although no satisfactory solutions were found to correct the misfit to the Rasch model, it is recommended that the response options of the brief MHQ be rescored, and that items 6, 9 and 10 be removed. The lack of unidimensionality indicates that the items do not represent the singular construct of hand disability and that totalling the scores of the brief MHQ does not provide a valid measure of hand disability for people with thumb osteoarthritis. The 37-item Michigan Hand Questionnaire may provide a better assessment of hand disability for patients with thumb osteoarthritis.

## Background

The Michigan Hand Questionnaire (MHQ) is a commonly used patient reported outcome for people with acute or chronic hand disorders that measures hand disability [1]. The MHQ consists of 37 items across 6 domains: overall hand function, activities of daily living, pain, work performance, aesthetics and satisfaction with hand function [2]. Its inclusion of questions regarding aesthetic appearances and satisfaction with hand function, which can be particularly important to patients with disfiguring hand conditions [2], distinguishes it from other upper limb questionnaires. The MHQ can also identify differences between both hands while adjusting for hand dominance [1] because the questions are answered for both the dominant and non-dominant hands [3]. Because the MHQ has been found to have redundant items in 4 of the 6 domains in a study that assessed the internal consistency of the MHQ in a sample of patients with rheumatoid arthritis [4], a brief version of the MHQ was created to improve efficiency in research [1]. In order to remove redundant items, item reduction and the consideration of clinical relevance were used to select which items to retain from the original MHQ [1]. The brief Michigan Hand Questionnaire (brief MHQ) retained 2 items from each of the 6 domains, resulting in a total of 12 items with no subscales [1].

While reducing the number of items can increase the response rate to a questionnaire [5], it can also increase variability and reduce accuracy [6]. During the development of the brief MHQ, Waljee et al. found that the mean scores of the brief MHQ were very similar to the mean scores of the original MHQ for a sample of 422 patients with rheumatoid arthritis, distal radius fracture, carpal tunnel, or thumb carpometacarpal osteoarthritis (correlation *r* = 0.99, *p* < 0.001) [1]. In this study, the data for the brief MHQ were extracted from the data collected by the MHQ, meaning that this data was paired. A comparison between the MHQ and the brief MHQ was also performed in patients with Dupuytren’s contractures [3]. The brief MHQ was highly correlated to the MHQ (*r* = 0.88). Unlike the study by Waljee et al., this study administered the MHQ and the brief MHQ separately, but it is not clear which questionnaire was administered first or if the questions in the questionnaires were given in random order [3]. For this reason, it is not clear if the participants were influenced by their answers on the MHQ when performing the brief MHQ. The brief MHQ has high test–retest reliability in sub-samples of patients with rheumatoid arthritis (ICC = 0.91) [1], and good internal consistency (Cronbach alpha = 0.88), good convergent validity with the Quick Disabilities of the Arm, Shoulder, and Hand (*r* = -0.82) in patients with Dupuytren’s contracture [3]. The brief MHQ was also validated in a variety of hand disorders as it correlated moderately with objective hand function measures (*r* = 0.36–0.41), and it was able to distinguish between known groups of different severity levels [1]. The brief MHQ was also found to be responsive for patients with distal radius fractures, carpal tunnel syndrome, rheumatoid arthritis and thumb osteoarthritis who received surgical interventions for their conditions [1].

Existing psychometric testing of the brief MHQ has used Classical Test Theory. The Classical Test Theory assumes that a) a test score is made up of a person’s true score and measurement error, b) measurement error is normally distributed with a mean of zero, and c) the error score and true score are unrelated [7]. Although Classical Test Theory is an acceptable and commonly used method, it can lack detail, and the test assumptions are often ignored [7]. This can compromise the psychometric test results leading to inaccurate conclusions. Unlike Classical Test Theory, Rasch analysis provides more detail about how a scale performs in addition to typical psychometric properties. Furthermore, assumption testing is built into the methods of Rasch analysis [8], and the assumptions will ensure that the resulting analysis improves the measurement properties of the tool [7]. To date, Rasch methods have not been applied to the brief MHQ, although Rasch analyses have been performed in the 37-item MHQ [9, 10]. Both studies identified that the MHQ had misfit to the Rasch model, identifying issues with the validity of the total MHQ score, but some subscale scores were found to have acceptable measurement properties [9, 10], which supports the continued use of the MHQ in research and clinical settings. Because the brief MHQ includes two items from each of the subscales after removing redundant items, but does not retain the subscale scores, it is important to determine if the total score of the brief MHQ is valid. If the total score of the brief MHQ is valid, the brief MHQ could be considered the preferred measure, in comparison to the 37-item MHQ, when the total score is required. If the total score of the brief MHQ is not valid, it will be important to revise the measure before continuing to use it in research and clinical settings. The Rasch analysis may be able to identify where the issues of validity would exist and how the brief MHQ could be revised, if required. Lastly, a Rasch analysis may be able to identify any issues with the scaling of the response options of the brief MHQ, and how the response option could be rescored.

The purpose of this study is to use Rasch measurement theory to assess the psychometric properties of the brief Michigan Hand Questionnaire in a sample of patients with thumb osteoarthritis, and to make suggestions for modifications to the questionnaire if issues with fit are identified.

## Methods

### Study design

This measurement study uses Rasch Measurement Theory to assess the psychometric properties of the brief MHQ and to identify the questionnaire’s fit to the Rasch model. This study used data collected from thumb osteoarthritis (OA) patients from clinics in the Netherlands [11]. All patients provided informed consent for the use of their anonymous data for research. Ethics approval was received for the data collection and the use of the data for research purposes from a local medical research ethics board in the Netherlands [11].

### Participants

Patients with hand and wrist conditions were treated at Xpert Clinic and Handtherapie Nederland, comprising a group of 18 clinics for hand surgery and hand therapy across the Netherlands. A total of 923 participants with thumb OA in one or both hands completed the Dutch language version of the MHQ at baseline as part of the routine measurements during usual clinical care [11]. The data were collected from September 2017 to June 2018 [11]. Demographic data, pain catastrophizing and illness perception were also collected from the participants. A more detailed explanation of the participant recruitment, and measurement and data collection procedures has been published elsewhere [11]. Demographic data has also been published elsewhere [9].

### Procedures

The counts and frequencies of the participant demographic data were obtained from the data structure summary on RUMM 2030 (RUMM Laboratory Pty Ltd, Perth, Australia). All the data reflect pretreatment values. The demographic data included age, sex, affected hand details (dominant hand, non-dominant hand or both hands), treatment type (surgical or non-surgical), pain catastrophizing measured by the Pain Catastrophizing Scale (scored from 0–52, with higher scores representing high levels of pain catastrophizing) [12], Illness Perception overall score (IPS) measured by the Brief Illness Perception Questionnaire (scored from 0–80, with higher scores representing a more negative perception of the illness) [13], and the type of work (heavy physical labour, moderate physical labour, light physical labour, or unemployed). Table 1 provides the coding categories used for each of the demographic variables. Because the items from the brief MHQ are identical to the corresponding items from the original 37 item MHQ [1], the 12 items of the brief MHQ were extracted from the original 37 item MHQ. The 12 items of the brief MHQ are listed in Table 2.

**Table 1:** A summary of the demographic data for the sample (*N* = 923), and the coding categories included in the Rasch analysis for each person variable [9]

Person variable	Coding categories	Number of persons (% of total sample *N* = 923)
Sex	Male	204 (22%)
	Female	719 (78%)
Age	Under 40	8 (1%)
	40 – 49 years	64 (7%)
	50 – 59 years	379 (41%)
	60 – 69 years	355 (38%)
	70 – 79 years	110 (12%)
	80 – 89 years	7 (1%)
Dominant hand treated	No	416 (45%)
	Yes	409 (44%)
	Ambidexter, one hand treated	30 (3%)
	Ambidexter, both hands treated	4 (0.4%)
	Both hands treated, not ambidexter	64 (7%)
Treatment choice	Non-surgical	579 (63%)
	Surgical	344 (37%)
Pain Catastrophizing Scale (0 – 52) [12]	Less than 15	589 (64%)
	15 – 23	199 (21.5%)
	24 – 38	121 (13%)
	39—52	14 (1.5%)
Illness perception overall score (0 – 80) [13]	0—10	7 (1%)
	11—20	37 (4%)
	21 – 30	110 (12%)
	31 -40	280 (30%)
	41 – 50	352 (38%)
	51 – 60	123 (13%)
	61 – 70	14 (2%)
	71 – 80	0 (0%)
Type of work	Unemployed	391 (42%)
	Light physical labor	194 (21%)
	Moderate physical labor	240 (26%)
	Heavy physical labor	98 (11%)

**Table 2:** The response frequencies for each of the 12 items of the brief MHQ. The item abbreviations are included in brackets; these abbreviations will be used to discuss the items in the paper

Item (Item abbreviation)	Response option frequency and percentage					Meaning of response option anchors	
	0	1	2	3	4	0	4
Overall, how well did your hand(s) work during the past week? (Satisfaction of hand function)	77 8%	256 28%	424 46%	150 16%	16 2%	Very good	Very poor
How was the sensation (feeling) in your hand(s) during the past week? (Feeling in hands)	92 10%	294 32%	363 39%	157 17%	17 2%	Very good	Very poor
How difficult was it for you to hold a frying pan during the last week? (Difficulty holding a frying pan)	90 10%	295 32%	269 29%	169 18%	100 11%	Not at all difficult	Very difficult
How difficult was it for you to button a shirt or blouse during the past week? (Difficulty buttoning shirt)	176 19%	301 33%	202 22%	149 16%	95 10%	Not at all difficult	Very difficult
How often were you unable to do your work in the past week because of problems with your hand(s)/wrist(s)? (Unable to work)	241 26%	156 17%	301 33%	190 20%	35 4%	Always	Never
How often did you take longer to do tasks in your work because of problems with your hand(s)/wrist(s)? (Slower work)	160 17%	101 11%	284 31%	293 32%	85 9%	Always	Never
Describe the pain in your hand(s)/wrist(s) in the past week? (Pain severity)	5 1%	71 7%	480 52%	340 37%	25 3%	Very mild	Very severe
How often did the pain in your hand(s)/wrist(s) interfere with your daily activities (such as eating or bathing)? (Pain during daily activities)	59 6%	116 13%	317 34%	349 38%	80 9%	Always	Never
I am satisfied with the look of my hand(s). (Satisfaction of appearance)	23 2%	237 26%	174 19%	139 15%	350 38%	Strongly agree	Strongly disagree
In the past week, the appearance of my hand(s) interferes with my normal daily activities. (Interference of appearance)	28 3%	22 2%	95 10%	222 24%	556 60%	Strongly agree	Strongly disagree
In the past week, how satisfied are you with the motion of your fingers? (Finger motion)	137 15%	266 29%	214 23%	237 26%	69 7%	Very satisfied	Very dissatisfied
In the past week, how satisfied are you with the motion of your wrist? (Wrist motion)	193 21%	271 29%	218 24%	180 19%	61 7%	Very satisfied	Very dissatisfied

Demographic data and the relevant MHQ data were used to determine the fit of the brief MHQ to the Rasch model. The Rasch analysis of the brief MHQ was performed using the RUMM 2030 software version (RUMM Laboratory Pty Ltd, Perth, Australia). The psychometric property testing of the brief MHQ followed Rasch analysis guidelines [14–18] and methods [19–21] that have been previously described in the literature. If issues with fit were identified at any point in the Rasch analysis, appropriate modifications were made to improve fit when possible. After making any changes required (i.e., rescoring or item deletion), an analysis of fit was performed, and the Rasch analysis was continued for the version of the brief MHQ with the best fit to the model. Based on previously established Rasch analysis methods, analyses including the analysis of fit, the Rasch analysis summary statistics, the calculations of the Person Separation Index and Cronbach’s alpha, and the t-tests for unidimensionality were performed for the original brief MHQ and any edited version of the model in order to make a comparison of key Rasch measurements [19].

### Statistical analysis

Although the brief MHQ contains 2 questions from each of the 6 subscales of the original MHQ, these 6 subscales are not maintained in the brief MHQ [1]. For this reason, the brief MHQ was analysed as the full questionnaire and did not need to be analysed by subscales. The following analysis steps were followed based on previously published methods [10, 14–21]. Bonferroni correction was used for the analyses when there was multiple testing [21].

1. *Likelihood Ratio test*: Because the brief MHQ has 5 response options per item, the items are considered polytomous, and before any other analyses can be performed, the polytomous model for the Rasch analysis must be determined. The Likelihood Ratio test was used to determine whether the Andrich Rating Scale Model or the Masters Partial Credit Model would be most appropriate for the analysis [14]. The partial credit model should be selected if the Likelihood Ratio tests is significant (*p* < 0.05), as this indicates that the differences between response options are unequal [21].
2. *Class intervals*: The class intervals were monitored throughout the different analyses. The class intervals are generated by the RUMM 2030 software by splitting the sample into classes based on the person variables. The goal is to have the class intervals as equal as possible [21].
3. *Analysis of fit summary statistics*: Before any manipulation of the brief MHQ (i.e., rescoring or item deletion), Rasch analysis summary statistics were assessed to determine the initial fit of the model (Power Analysis of Fit and the item-trait interaction) as well as the mean item location, the mean person location and the associated fit residuals. A large total-item Chi Square (χ^2^) value and a significant item-trait interaction identifies a breach of the requirement of invariance, indicating misfit of the brief MHQ to the model [19]. The mean item location and mean person location are expected to be 0 with a standard deviation of 1 because the item-person data is transformed to approximate a normal distribution [19].
4. *Brief MHQ response frequencies*: The category response frequencies for each item were assessed to determine how often each response option was endorsed by this sample. Items with response options that were endorsed less than 10 times were flagged to be addressed in the rescoring of the items. It is recommended that there are at least 10 endorsements for each response option of an item [22]. Floor and ceiling effects were also assessed by determining if the minimum or maximum response options were endorsed by more than 15% of the sample, which is an arbitrary cutoff point inspired by the analyses performed by Beaton et al. (2010) and Terwee et al. (2007) [23, 24]**.**
5. *Threshold distributions*: The threshold map was first assessed to identify any disordered thresholds. The category probability curves were then visually inspected to get more detail on the disordered thresholds in order to identify which response options were difficult to distinguish. In order to address the disordered thresholds, the items were rescored by collapsing the response options [21]. Once the items were rescored, the threshold map was visually inspected to confirm that the disordered thresholds were fixed.
6. *Fit to the Rasch model*: After rescoring any required items, the analysis of fit summary statistics (identified in step 3) were reassessed to determine the fit of the rescored brief MHQ.
7. *Individual person fit*: The mean person fit residuals and the number of persons with excessive fit residuals (outside the range of -2 to + 2) were identified [25]. The Person-Item Threshold distribution was visually inspected to identify if the item difficulty spread appeared to match the spread of the person ability in order to determine if the questionnaire was well-targeted towards patients with thumb OA [26]. Analysis of Variance (ANOVA) tests of the Person-Item Threshold Distributions specific to each person factor (i.e., the demographic variables such as sex) were performed to determine if group differences existed in the mean abilities of the subgroups (i.e. males and females).
8. *Individual item fit*: The 12 rescored items of the brief MHQ were assessed for fit residuals. Items with fit residuals outside of the acceptable range of -2.5 and + 2.5 and a significant *p*-value (Bonferroni correction for the significance threshold of α = 0.05 for multiple analyses) were identified as not fitting the Rasch model [20]. Items that did not fit the Rasch model were removed and the analysis of fit summary statistics (identified in step 3) were reassessed to determine the fit of the rescored brief MHQ.
9. *Differential item functioning (DIF)*: DIF was assessed, both visually and statistically, through ANOVA, using the Item Characteristic Curves for each item. The items were assessed across the different class intervals for each person factor (i.e., sex, age, hand dominance, treatment choice, pain catastrophizing, illness perception, and type of work; see Table 1). Uniform DIF occurs when the between-group differences are consistent across the latent trait (parallel Item Characteristic Curves), whereas non-uniform DIF occurs when the between-group differences vary across the latent trait (non-parallel Item Characteristic Curves) [27]. Both uniform and non-uniform DIF were analysed by assessing the main person factor effect and the interaction effect of the person factor and the class interval, respectively. Significant findings, based on a Bonferroni correction for the significance threshold of α = 0.05 for multiple analyses, identified DIF [19]. If uniform DIF is detected, the item can be split into separate items based on the specific demographic variable [19]. If non-uniform DIF is detected, the item cannot be split; thus, item deletion is the only way to correct the issue, although this can affect the validity of the questionnaire [27]. Because identified DIF that cannot be remedied violates the requirement of unidimensionality, this was considered as a test of unidimensionality [19].
10. *Person Separation Index (PSI) and Cronbach’s alpha*: The PSI and Cronbach’s alpha were calculated as a measure of reliability. An explanation of the PSI can be found elsewhere [20].
11. *Unidimensionality and Local Independence*: Along with the assessment of DIF, unidimensionality was assessed by performing the Principal Component Analysis. Unidimensionality and local dependence were assessed before any modification to the items, and after modification were made [10]. The items that positively loaded and negatively loaded on the first principal component were used as the two subsets of the items for the paired t-tests. In order to meet the requirement of unidimensionality, the frequency of t-tests that are significant (*p* < 0.05) must be below five percent [10, 21]. Local independence was assessed by inspecting the correlations of the item residuals for any patterns or high correlations [19] which are generated using the Principal Component Analysis. A correlation value larger than 0.2 points above the average correlation value was used to identify highly correlated item residuals, and thus the indicator of local dependence [28, 29]. If local dependence is identified, the items that are highly correlated can be combined into subtests in order to correct the local dependence [14].

## Results

### Demographics

The data from 923 completed MHQ assessments were analysed in this study. The sample included 204 (22%) males and 719 (78%) females. Table 1 [9] includes a summary of the demographic data as well as the coding categories for each person variable in the Rasch analysis.

### Rasch analysis summary statistics

The data from all 923 participants were included in the analysis as no extreme scores were identified. The likelihood ratio test was significant; thus, the unrestricted partial credit model was deemed most appropriate for this analysis. When assessing the class interval distribution, 5 class intervals were found to allow for the most even distribution between intervals (164 – 203). The mean item location was set to 0.00 with a resulting standard deviation (SD) of 0.47. The mean item fit residual was 1.25 (SD = 6.34). The sample appeared to be an appropriate match to the scale as the mean persons location was -0.03 (SD = 0.57). The mean person fit residual was -0.13 (SD = 1.26). The overall Power of Analysis of Fit rated the fit as “Good”, although the item-trait interaction was found to be significant (total-item χ^2^ = 1312.5, df = 48, *p* < 0.0001), indicating misfit of the model.

### Brief MHQ response frequencies

All response options for the 12 items of the brief MHQ were endorsed. For the item “Describe the pain in your hand(s)/wrist(s) in the past week?”, only 5 individuals answered this question as “very mild” (Table 2), which is less than the recommendation for at least 10 endorsements for a category [22]. The items “Satisfaction of appearance”, “Unable to work”, and “Slower work” appeared to have a ceiling effect as the highest response option (38%, 26% and 17% of participants endorsed the response option representing the highest level of hand disability for each item respectively) for the items were highly endorsed compared to the other options [30] (Table 2). The items “Difficulty buttoning shirt”, “Interference of appearance”, and “Wrist motion” appeared to have a floor effect as the lowest response option (19%, 60%, 21% of participants endorsed the response option representing the lowest level of hand disability for each item respectively) for the items were highly endorsed [30] (Table 2). The percent of participants endorsing the minimum and maximum response options for these items exceeds 15%, identifying floor effects in 4 items and ceiling effects in 2 items [23, 24].

### Thresholds

**Fig. 1:**
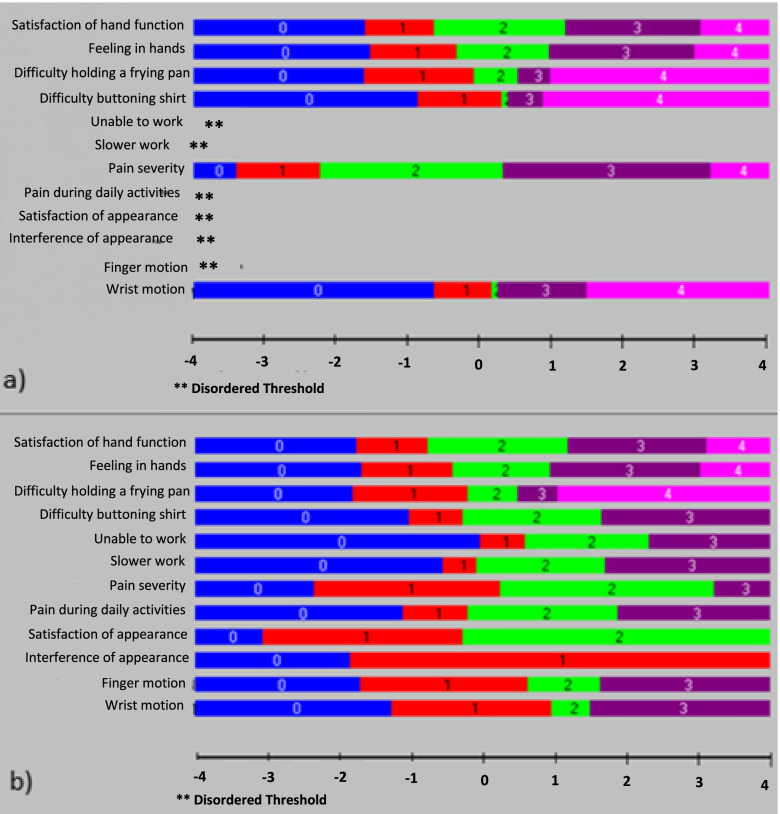
a The threshold map for original 12 items brief MHQ. b The threshold map for the rescored brief MHQ. Each colour bar represents a response option; blue represents response option 0, red represents response option 1, green represents response option 3, purple represents response option 4, and pink represents response option 5. Disordered thresholds are represented by **

When analysing the ordering of the thresholds, six of the 12 items of the brief MHQ had disordered thresholds (Fig. 1a). By referencing the category probability curves, these six items were re-scored by collapsing response categories in order to achieve proper ordering of the thresholds. Upon further inspection of the category probability curves for each item, difficulty in discriminating between response options 1, 2 and 3 was also found in two other items. These items were rescored by collapsing response option 2. Lastly, due to the lack of endorsement of response option “very mild” for the item “Pain severity”, this response option was collapsed [22] (see Table 3 for the rescoring of the items and Fig. 1b for the rescored threshold map). After rescoring the items, 3 class intervals were deemed more appropriate than the original 5 class intervals as there was a more even distribution (294 – 327). Three class intervals were used for the remaining analyses.

**Table 3:** The rescoring of the disordered thresholds. Items 1, 2 and 3 did not require rescoring and reflect the original scoring system

Item (Item abbreviation)	Scoring	0	1	2	3	4
Overall, how well did your hand(s) work during the past week? (Satisfaction of hand function)	Original scoring	0	1	2	3	4
How was the sensation (feeling) in your hand(s) during the past week? (Feeling in hands)	Original scoring	0	1	2	3	4
How difficult was it for you to hold a frying pan during the last week? (Difficulty holding a frying pan)	Original scoring	0	1	2	3	4
How difficult was it for you to button a shirt or blouse during the past week? (Difficulty buttoning shirt)	Rescored	0	1	2	2	3
How often were you unable to do your work in the past week because of problems with your hand(s)/wrist(s)? (Unable to work)	Rescored	0	0	1	2	3
How often did you take longer to do tasks in your work because of problems with your hand(s)/wrist(s)? (Slower work)	Rescored	0	0	1	2	3
Describe the pain in your hand(s)/wrist(s) in the past week? (Pain severity)	Rescored	0	0	1	2	3
How often did the pain in your hand(s)/wrist(s) interfere with your daily activities (such as eating or bathing)? (Pain during daily activities)	Rescored	0	0	1	2	3
I am satisfied with the look of my hand(s). (Satisfaction of appearance)	Rescored	0	1	1	2	2
In the past week, the appearance of my hand(s) interferes with my normal daily activities. (Interference of appearance)	Rescored	0	0	0	1	1
In the past week, how satisfied are you with the motion of your fingers? (Finger motion)	Rescored	0	1	1	2	3
In the past week, how satisfied are you with the motion of your wrist? (Wrist motion)	Rescored	0	1	1	2	3

### Fit to the Rasch model

After rescoring the items with disordered thresholds and response categories that were difficult to discriminate, there was misfit of the brief MHQ to the Rasch model as the item-trait interaction was significant (total-item χ^2^ = 522.7, df = 24, *p* < 0.0001).

### Individual person fit

**Fig. 2:**
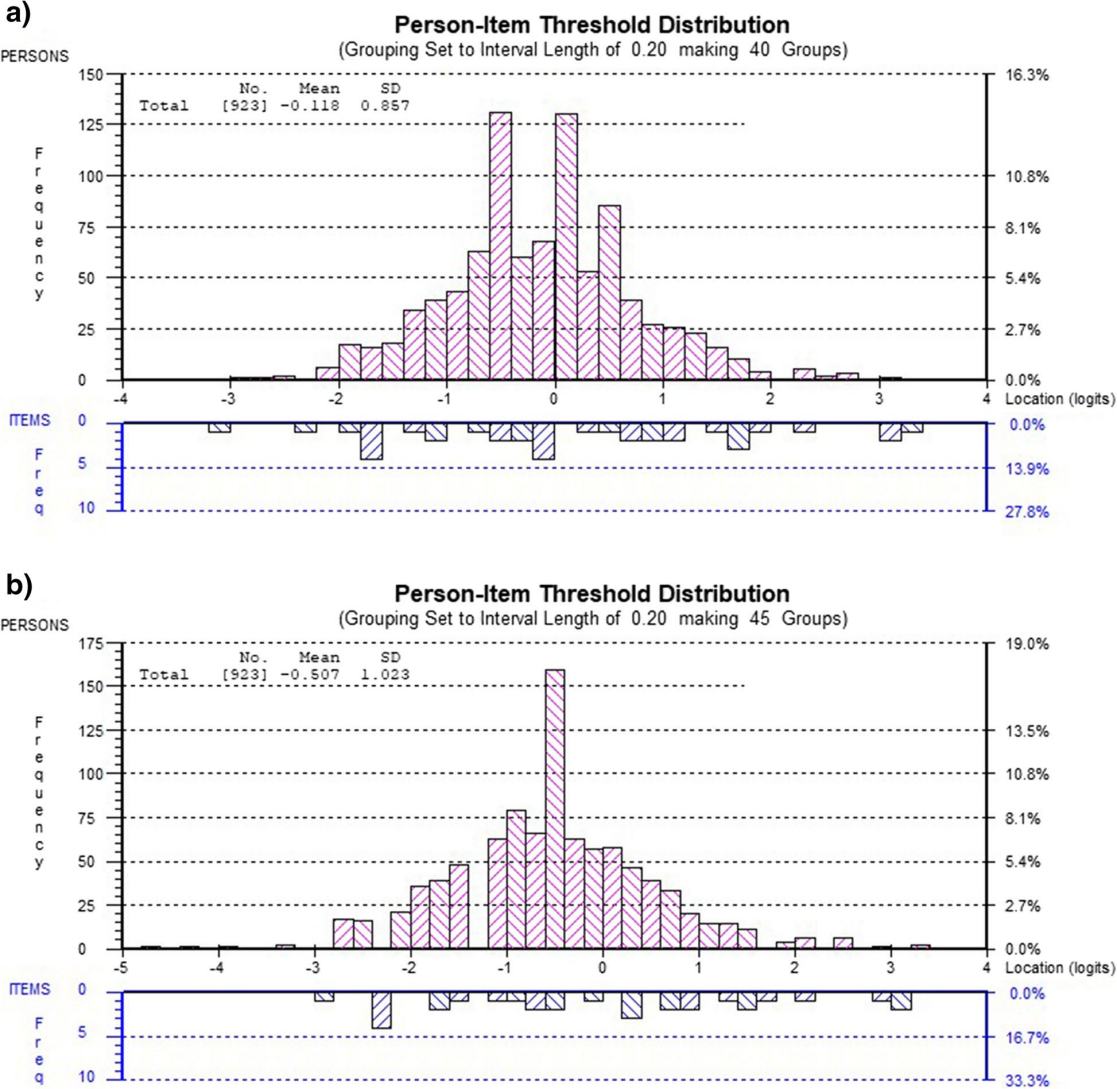
a The Person-Item Threshold Distribution of the rescored 12-item brief MHQ. b The Person-Item Threshold Distribution of the 9-item brief MHQ. The Person-Item Threshold Distribution illustrates person ability on the top portion of the graph, and item difficulty on the bottom portion of the graph. The vertical axis represents frequency of participants and items. The horizontal axis represents the ability in logits measured by the questionnaire, which is hand disability for the brief MHQ. Negative scores (on the left) represent less hand disability and positive scores (on the right) represent more hand disability. Each bar on the graph represents the frequency of participants and the frequency of items at each level of hand disability. This graph is inspected visually in order to assess how well the item difficult and person ability match

After rescoring the 12 items of the brief MHQ, the mean person location was -0.12 (SD = 0.86) with a mean person fit residual of -0.31 (SD = 1.22). Eighty-nine individuals (9.6% of the total sample) had person fit residuals outside of the acceptable range of -2 to + 2. Based on visual inspection of the Person-Item Threshold Distribution (Fig. 2a), the item difficulty spread appeared to fit the individual ability spread fairly well, although there were visible gaps at both extremities of the item difficulty spread (visually represented at the extremities of the curve on the graph in Fig. 2a). This supports the floor and ceiling findings identified from the item response frequencies. When assessing the Person-Item Threshold Distribution based on the person factors, sex, treatment choice, Pain Catastrophizing Scale score and Illness Perception score, all had significant differences amongst mean locations of the categories (Table 4).

**Table 4:** Findings of the analysis of variance of the Person-Item Threshold Distributions for each person variable

Person variable	Rescored 12-item Brief MHQ p-value	Variable categories	Category mean (logits)	Category standard deviation (logits)	Description of the direction of the differences
Gender	0.0004*	Male	-0.31	0.81	Men reported less hand disability then women
		Female	-0.065	0.86	
Age	0.6983	Under 40	-0.063	0.88	No differences
		40 – 49	0.014	1.01	
		50 – 59	-0.10	0.86	
		60 – 69	-0.17	0.85	
		70 – 79	-0.10	0.80	
		80 – 89	-0.036	0.75	
Dominant hand treated	0.2748	No	-0.080	0.85	No differences
		Yes	-0.12	0.86	
		Ambidextrous, one hand treated	-0.10	0.95	
		Ambidextrous, both hands treated	-0.10	0.59	
		Both hands treated, not ambidextrous	-0.34	0.84	
Treatment choice	< 0.0001*	Non-surgical	-0.30	0.82	Non-surgical patients reported less hand disability then surgical patients
		Surgical	0.19	0.83	
Pain Catastrophizing Scale (0–52) [12]	< 0.0001*	Less than 15	-0.34	0.79	Patients with lower pain catastrophizing scores reported less hand disability then patients with higher pain catastrophizing scores
		15 – 23	0.077	0.73	
		24 – 38	0.47	0.81	
		39 – 52	1.44	1.00	
Illness perception overall score (IPS) (0–80) [13]	< 0.0001*	0 – 10	-1.42	0.98	Patients with lower IPS reported less hand disability then patients with higher IPS
		11 – 20	-0.87	0.68	
		21 – 30	-0.70	0.76	
		31 – 40	-0.25	0.78	
		41 – 50	0.043	0.71	
		51 – 60	0.42	0.90	
		61 – 70	0.89	0.86	
		71 – 80	0.0	0.0	
Type of work	0.3151	Unemployed	-0.061	0.83	No differences
		Light physical labor	-0.19	0.88	
		Moderate physical labor	-0.15	0.87	
		Heavy physical labor	-0.11	0.87	

^*^identifies significant *p*-value

### Individual item fit

Three items with a fit residual outside of the acceptable range of -2.5 and + 2.5 and a significant Bonferroni corrected *p* < 0.01 based on the number of analyses performed were identified: item 9 “Satisfaction of appearance” (fit residual = 12.05, *p* < 0.0001), item 10 “Interference of appearance” (fit residual = 8.46, *p* < 0.0001), and item 6 “Slower work” (fit residual = -3.06, *p* = 0.0001) (Table 5). Items with significant misfit that exceeds ± 3 can be considered to provide strong evidence of misfit to the Rasch model [28]; thus, these items were removed. The 9 remaining items had acceptable fit residuals within -2.5 and + 2.5 and had non-significant p-values (Table 5). The mean item location and standard error can be found in Table 5.

**Table 5:** Item fit statistics for the rescored 12-item brief MHQ and the 9-item brief MHQ

Item	Rescored 12-item brief MHQ			9-item brief MHQ		
	Mean item location	Standard error	Fit Residual (p-value)	Mean item location	Standard error	Fit Residual (p-value)
Satisfaction of hand function	0.458	0.043	-1.337 (0.015)	0.116	0.045	0.445 (0.011)
Feeling in hands	0.479	0.041	0.045 (0.543)	0.139	0.043	1.716 (0.021)
Difficulty holding a frying pan	-0.105	0.036	-2.909 (0.041)	-0.477	0.037	-0.938 (0.125)
Difficulty buttoning a shirt	0.129	0.042	-2.379 (0.027)	-0.232	0.044	-0.811 (0.279)
Unable to work	0.975	0.044	-2.545 (0.0001)	0.651	0.046	1.058 (0.183)
Slower work	0.370	0.041	-4.994 (< 0.0001)^a^	Removed	Removed	Removed
Pain severity	0.389	0.055	-1.723 (0.004)	0.025	0.057	-0.327 (0.036)
Pain during daily activities	0.201	0.044	-3.068 (0.0001)	-0.163	0.045	-1.032 (0.008)
Satisfaction of appearance	-1.664	0.062	12.050 (< 0.0001)^a^	Removed	Removed	Removed
Interference of appearance	-1.833	0.090	7.158 (< 0.0001)^a^	Removed	Removed	Removed
Finger motion	0.196	0.047	-0.149 (0.559)	-0.149	0.049	1.220 (0.281)
Wrist motion	0.404	0.046	-0.087 (0.485)	0.090	0.0480	1.086 (0.144)

^a^ identifies large significant fit residuals; these items were removed

After deleting three items from the brief MHQ, the mean person location changed to -0.50 (SD = 1.01) with a person fit residual of -0.32 (SD = 1.20). Also, the visual inspection of the 9-item brief MHQ Person-Item Threshold Distribution (Fig. 2b) identified that the item difficulty spread did not match the person ability spread as well as the 12-item brief MHQ did as the small floor and ceiling effect appeared more noticeable at both extremities of the curve on the graph. After deleting the three items, the 9-item brief MHQ still did not fit the Rasch model, as the item-trait interaction was still significant (χ^2^ = 49.6, df = 18, *p* = 0.0001).

### Differential item functioning

In order to confirm the deletion of the 3 items (item 9 “Satisfaction of appearance”, item 10 “Interference of appearance”, and item 6 “Slower work”), DIF was assessed for these items across all person variables. The ANOVA analyses and visual inspection found significant non-uniform DIF across many of the person variables. The ANOVA analysis also identified significant misfit for these items (p < 0.0001). These items were thus removed from the analysis and the DIF was repeated for the 9-item brief MHQ.

Seven of the 9 remaining items had some uniform DIF across different variables, as per the ANOVA of the main person effect. No significant interaction effects of the person factor and the class interval were identified, meaning that no non-uniform DIF was found, as per ANOVA. Upon visual inspection of the item characteristic curves for each item and person variables, the DIF appeared to be non-uniform for most of the items with DIF. Upon inspection of the means of the observed scores across the class intervals for each item that had DIF across person variables, the differences were not found to be even across the class intervals, identifying that the DIF was non-uniform for most items.

A summary of the items with DIF can be found in Table 6. Often, items with highly significant DIF can cause artificial DIF in other items. Because the item “Describe the pain in your hand(s)/wrist(s) in the past week” had the most significant DIF across three person variables, this item was deleted [27]. This deletion did not remedy the DIF found in the other items and actually worsened the non-uniform DIF of other items. Even after the sequential deletion of other items with significant DIF, the DIF in other items did not improve and created new non-uniform DIF in other items and across other demographic variables. Because deleting items due to non-uniform DIF can be found to cause issues with the validity of the questionnaire [27], none of the items with non-uniform DIF were removed. Instead, the non-uniform DIF indicated that the brief MHQ is not unidimensional. The item “Feeling in hand” was found to have uniform DIF for the treatment choice person variable. Although splitting this item based on treatment choice (surgery or therapy) did not improve the fit of the brief MHQ to the Rasch model, no additional issues such as DIF or item misfit were created when this item was split. Splitting the item “Feeling in hand” based on treatment choice corrected the uniform DIF issue. When splitting the item “Difficulty holding frying pan” by sex and the item “Finger motion” was considered, additional non-uniform DIF was created, thus these items were retained without splitting.

**Table 6:** Results of the assessment of DIF for each of the 9 remaining items of the brief MHQ, across each person variable. DIF was assessed in three ways: ANOVA, visual inspection of the DIF graphs and by inspecting the mean scores across the class intervals

Item	Person variable	Significant main person effect *p*-value	Visual inspection of the item characteristic curve	Inspection of the category means scores across the class intervals
How was the sensation (feeling) in your hand(s) during the past week?	Treatment choice	0.0005	Uniform DIF	Mean differences between categories are relatively even across class intervals
How difficult was it for you to hold a frying pan during the last week?	Sex	0.0003	Non-uniform DIF (DIF graph lines intersect)	Mean differences between categories are uneven across class intervals
How difficult was it for you to button a shirt or blouse during the past week?	Sex	0.0008	Uniform DIF	Mean differences between categories are relatively even across class intervals
How often were you unable to do your work in the past week because of problems with your hand(s)?	PCS	0.001	Non-uniform DIF (DIF graph lines intersect)	Mean differences between categories are uneven across class intervals
Describe the pain in your hand(s)/wrist(s) in the past week?	PCS	< 0.0001	Non-uniform DIF (DIF graph lines intersect)	Mean differences between categories are uneven across class intervals
	IPS	< 0.0001	Non-uniform DIF (DIF graph lines intersect)	Mean differences between categories are uneven across class intervals
	Treatment choice	< 0.0001	Non-uniform DIF	Mean differences between categories are uneven across class intervals
How often did the pain in your hand(s)/wrist(s) interfere with your daily activities (such as eating or bathing)?	Treatment choice	0.0003	Non-uniform DIF (DIF graph lines intersect)	Mean differences between categories are uneven across class intervals
In the past week, how satisfied are you with the motion of your fingers?	Treatment choice	0.001	Uniform DIF	Mean differences between categories are uneven across class intervals

**Table 7:** Summary statistics of the original brief MHQ, the rescored 12-item brief MHQ and the 9-item brief MHQ

	Item-trait interaction	Item location mean (SD)	Item fit residual (SD)	Person location mean (SD)	Person fit residual (SD)	Person Separation Index	Cronbach’s alpha	Unidimensionality (% of t-test < 0.05)
Original 12-item brief MHQ	χ^2^ = 1312.5 df = 48 *p* < 0.0001^a^	0.0 (0.47)	1.25 (6.34)	-0.03 (0.57) No extremes (*N* = 923)	-0.13 (1.26)	0.72	0.71 (*N* = 921)	11.81%^b^
Rescored 12-item brief MHQ	χ^2^ = 522.7 df = 24 *p* < 0.0001^a^	0.0 (0.86)	0.005 (4.83)	-0.12 (0.86) No extremes (*N* = 923)	-0.31 (1.22)	0.78	0.78 (*N* = 921)	9.97%^b^
9-item brief MHQ	χ^2^ = 49.6 df = 18 *p* = 0.0001^a^	0.0 (0.32)	0.27 (1.06)	-0.50 (1.01) 2 extremes (*N* = 923)	-0.32 (1.20)	0.79 with extremes 0.79 without extremes	0.79 with extremes 0.79 without extremes (*N* = 921)	10.2%^b^

^a^identifies misfit to the model and a breach of the requirement of invariance

^b^identifies a breach of the requirement of unidimensionality

### Person separation index (PSI)

For the 9-item Brief MHQ (after 3 items were removed), the PSI was 0.79, whether or not the 2 extreme persons were included in the analysis. In order to calculate Cronbach’s alpha for the 9-item Brief MHQ, 2 persons with missing data were removed (adjusted sample size = 921) and one extreme person was identified. The Cronbach’s alpha was 0.79, whether or not the extreme person was included in the analysis.

### Unidimensionality and location independence

The 9-item brief MHQ was assessed for local independence by inspecting the correlations of the item residuals. Local dependence was found for the item “Difficulty buttoning shirt” and the item “Wrist motion” (correlation = -0.327), for the item “Feeling in hands” and the item “Pain during daily activities” (correlation = -0.310), for the item “Feeling in hands” and the item “Difficulty holding a frying pan” (correlation = -0.292), and for the item “Satisfaction of hand function” and the item "Pain during daily activities” (correlation = -0.294). When highly correlated items were combined to create subtests, new local dependence was identified between the subtest items and other items of the brief MHQ. The combined items had large item fit residuals (> ± 0.292). Because the combined subtests items did not fix the issue of local dependence and had large fit residuals, the items “Difficulty buttoning shirt”, “Wrist motion”, “Feeling in hands”, “Pain during daily activities”, “Difficulty holding a frying pan” and “Satisfaction of hand function” were retained as individual items.

The t-test of the items that positively and negatively loaded on the first principal component found that 94 t-test analyses (10.2%) were significant at *p* < 0.05, indicating that the brief MHQ is not unidimensional. The results of the tests for unidimensionality as well as the summary statistics for the original 12-item brief MHQ, the rescored 12-item MHQ and the 9-item brief MHQ can be found in Table 7.

## Discussion

Our Rasch analysis of the brief MHQ, using data from the 37-item MHQ from a sample of individuals with thumb OA, identified that there was misfit to the Rasch model, that the progression of the scoring did not follow an orderly progression for 9 of the 12 items, and that the questionnaire was not unidimensional. Similar to the study by Wehrli et al. in 2016, which found that the brief MHQ had good internal consistency (Cronbach alpha = 0.88) as measured by the Classical Test Theory [3], this Rasch analysis identified that the original brief MHQ had acceptable reliability (PSI = 0.72, Cronbach’s alpha = 0.71) based on an acceptable reliability score of 0.7 or higher [14]. Unlike a past study that used Classical Test Theory psychometric testing to validate the brief MHQ in patients with thumb OA, which found that the brief MHQ had acceptable construct validity with the MHQ (*r* = 0.99) [1], this Rasch analysis found that the brief MHQ is not suitable for assessing hand disability in patients with thumb OA. Because the MHQ and the brief MHQ were both created based on Classical Test Theory psychometric testing [1], the brief MHQ was not expected to fit the Rasch model without adjusting the response options of some items [20]. However, we were unable to find a satisfactory solution. Similar issues were identified in the Rasch analysis of the 37-item MHQ in a sample of patients with thumb OA, as 11 of the 37 items required rescoring but the 37-item MHQ still had misfit to the Rasch model [9].

The Rasch analysis identified several issues with the response options of the brief MHQ. First, the response options of certain items were not proportionally endorsed. For example, the item “Pain severity” only had 5 participants respond that their pain was “very mild”, whereas 480 participants responded that their pain was “moderate”. Because the sample in this study has thumb OA, a condition that is likely to cause pain during daily activities [31, 32], it is likely that the response option “very mild” for the “Pain severity” item was not appropriate for this thumb OA population. Moreover, ceiling affects were seen for items “Satisfaction of appearance”, “Unable to work”, and “Slower work” as the response option representing the highest level of disability was highly endorsed (350, 241 and 160, respectively). For the item “I am satisfied with the look of my hand(s)”, 350 participants (38%) selected “strongly disagree”, which is expected as deformities or enlargements are common in thumb OA [31, 32]. Floor effects were also identified in 3 items, “Difficulty buttoning shirt”, “Interference of appearance”, and “Wrist motion”. These floor and ceiling effects identify that the item difficulty may not be suitable for all patients with thumb OA [30]. It is possible that the brief MHQ may require both easier items and more difficult items in order to target patients with thumb OA who have severe hand disability and minor hand disability. The Rasch analysis also identified that the pattern of responses did not follow a predictable progression relative to the person abilities, resulting in the need to rescore items. For 9 of the 12 items, rescoring by combining response options was necessary. For example, dichotomous response options (“Agree” or “Disagree”) were found to be the most appropriate for the item “Interference of appearance”. In Classical Test Theory, the ideal number of response options ranges from 5 to 10, as scales with 4 response options or less generally have weaker reliability and validity [33]. Yet, in this study, reducing the number of response options for 75% of the brief MHQ items improved the reliability of the questionnaire (Cronbach’s alpha improved from 0.71 to 0.78). Similar findings were identified in the Rasch analysis of the 37-item MHQ in patients with hand and wrist conditions as this study rescored 10 items to a 3-point Likert scale and 11 items to a 5-point Likert scale [10].

Although rescoring the items of the brief MHQ did slightly improve the overall fit to the Rasch model, the item fit residual standard deviation was still high (see Table 6), indicating that items with large residual fits exist. Two items, “Satisfaction of appearance” and “Interference of appearance” had large and significant fit residuals (12.05, *p* < 0.0001, and 8.46, *p* < 0.0001, respectively). Another item, “Slower work”, was also found to have a significant fit residual (fit residual = -3.06, *p* = 0.0001) outside the acceptable range of -2.5 to + 2.5 [20]. These three items present strong evidence of misfit as the significant fit residuals exceed ± 3 [28]. Similarly, in a Rasch analysis of the 37-item MHQ in patients with thumb OA, the aesthetics subscale and work performance subscale did not fit the Rasch model, as there were issues identified with the items in these subscales [9]. In this Rasch analysis of the brief MHQ, “Satisfaction of appearance”, “Interference of appearance” and “Slower work” were also assessed for DIF to determine if uniform DIF across person variables were the cause of the large residuals. Because significant non-uniform DIF was observed across multiple person variables, and because the DIF ANOVA analyses identified significant misfit of the items, these items were deleted. When large item fit residuals and non-uniform DIF are identified, the only method of correction is to delete the item [21]. Not all items with non-uniform DIF were deleted from the analysis when they had appropriate fit to the Rasch model as deleting items can negatively impact the content validity of the questionnaire [27].

The brief MHQ was created based on the original 37 item MHQ, and the two questionnaires were identified to be highly correlated [1, 3]. By removing items of the brief MHQ, it is possible that the brief MHQ may no longer be similar enough to the original 37 item MHQ. For example, the items “Satisfaction of appearance” and “Interference of appearance” were the two items selected to represent the MHQ’s Aesthetic domain in the brief MHQ [1]. As these two items were removed in this Rasch analysis due to misfit and non-uniform DIF, the Aesthetic domain was no longer represented in the Rasch version of the brief MHQ. Deleting items has clinical implications as it is no longer known if the brief MHQ can be used interchangeably with the 37 item MHQ. The 9-item brief MHQ would also not provide clinicians with any details regarding the impact of thumb OA on the appearance of the hand, which can be important to consider for patients with disfiguring hand conditions [2]. It is possible that appearance can limit hand function as that both relate to joint deformities. In this case, the 37-item MHQ may be a better option for assessing hand disability in patients with thumb OA because the aesthetics subscale was found to have acceptable fit to the Rasch model in a sample of patients with hand and wrist conditions [10] and in a sample of patients with rheumatoid arthritis [34] after modifications were made. Future research would be needed to assess the correlations between the reduced item brief MHQ and the 37-item MHQ in a sample of individuals with thumb OA. The issue about whether the aesthetic items are critical relates to the content validity of the questionnaire. It is possible that our findings relate to an underlying problem with aesthetics not fitting in with hand function, at least for a majority of people with thumb OA. In this case, measurement of the construct of aesthetics and appearance would be appropriately measured separately. However, removal of the items does mean that the comparability of the scores can no longer be assumed.

Although the rescoring and the deletion of items with poor fit did improve the overall fit and the reliability of the brief MHQ, there was still misfit to the model, local dependence of some of the remaining items, and a lack of unidimensionality. It is possible that local dependence and a lack of unidimensionality are a result of how the brief MHQ was developed because they are common when questionnaires include items from different domains that represent a latent construct [29]. The lack of unidimensionality means that it is not valid to sum the responses from the brief MHQ to provide a total score [19, 25]. When clinicians use the total score of the brief MHQ to assess the health status of patients with hand disabilities such as thumb OA, this total score would not be valid, and thus it may not provide an accurate assessment of health status. In a Rasch analysis of the 37-item MHQ, lack of unidimensionality of the scale and misfit to the Rasch model were also identified [10]. Because the 37-item MHQ has 6 subscales, each subscale was further assessed for unidimensionality. Each subscale was found to be unidimensional, and 3 subscales (activities of daily living, aesthetics and satisfaction) were found to fit the Rasch model, identifying that these subscales can provide valid total scores [10]. In another Rasch analysis of the 37-item MHQ, three subscales (overall hand function, pain and unilateral activities subscales) were found to be unidimensional and to fit the Rasch model [9]. On the other hand, a study by Jayaram et al. found that with some modifications to the items, the MHQ and all 6 of the subscales fit well to the Rasch model [34]. When comparing the Rasch analysis findings of the 37-item MHQ [9, 10, 34] and the brief-MHQ, the 37-item MHQ would be more valid and useful for clinicians because the subscale scores would provide valid measures of hand disability. Moreover, in this Rasch analysis of the brief MHQ identified local dependence for two sets of items: the items “Difficulty buttoning shirt” and “Wrist motion”, and for the items “Feeling in hands” and “Pain during daily activities”. Although the items that were correlated represented different domains from the original 37 item MHQ, it is likely that they measure similar constructs. These correlation values identify that the items are linked and that the response given for one question will influence the response given for another question [15]. For example, poor feeling in the hands and higher levels of pain are concurrent symptoms for people with more severe hand disability due to thumb OA [31, 32].

A strength of the brief MHQ is that the range of item difficulty was similar to the range of person ability for this sample of participants with thumb OA, whether the Person-Item Threshold Distribution of the rescored 12-item brief MHQ or the 9-item brief MHQ were assessed, although there were visible gaps at both extremities of the item difficulty range. The mean person location was close to zero (-0.12 for the rescored 12 item-brief MHQ, -0.50 for the 9-item brief MHQ), indicating the proper targeting, although the negative mean person locations identify that the sample of participants with thumb OA had slightly less hand disability than the brief MHQ items intended to measure. The small floor effects in 4 items and the large ceiling effects in 2 items do identify that the brief MHQ may require additional items that target patients with lower levels and higher levels of hand disability [30]. The Person-Item Threshold Distributions identified that sex, treatment choice, Pain Catastrophizing Scale score and Illness Perception overall score had significant between-group differences when comparing the mean person location for the subgroups of the person variable. For example, males were found to report significantly less hand disability than women do, whereas participants with higher pain catastrophizing would have more self-reported hand disability than those with low pain catastrophizing. This could mean that these person variables have effects on how participants respond to the items of the brief MHQ, but due to the DIF identified in the analysis, it is unclear if these significant differences are meaningful or a result of error. These findings, along with the presence of DIF for these same four person variables, indicate that there is item bias. Item bias occurs when the items in a scale do not function comparably for all individuals at the same level of item-difficulty, as the responses are influenced by another characteristic [25]. Moreover, age, the hand(s) affected by thumb OA and the type of work demands, did not influence how the participants would score on the brief MHQ. For example, whether the participants had thumb OA in their dominant, non-dominant or both hands, the participants did not score differently on the brief MHQ. This finding, along with the finding that no DIF existed for the affected hand across the items of the brief MHQ have clinical and research implications. This means that no risk of bias exists in any of the items as well as in the full questionnaire when comparing individuals with dominant thumb OA, non-dominant thumb OA or thumb OA in both hands. Because the MHQ was created to assess differences in disability between both hands [1], it is important for clinicians and researchers to know that between hand differences identified are due to true difference rather than item response bias.

A limitation of the study was that the original data collection was not done with the brief MHQ; instead, the data was collected using the MHQ, which contains 37 questions. Only the data from the 12 relevant items were used for this Rasch analysis. Although Rasch analysis requires items to be independent of each other, the answers of the items that were not included in this analysis may have had an influence on the way the participants responded to the 12 items included in this analysis [35]. It is possible that the participants may have responded differently to certain items if they had only completed the brief MHQ rather than the 37 item MHQ. However, given the fact that the brief MHQ items represent the same items from the same domains subscales as the full MHQ, we think this was unlikely to have had any substantial effect on our findings. A future study employing a Rasch analysis with a sample of patients that answered only the items of the brief MHQ may confirm this. Because secondary data was used for this study, the Rasch analysis was limited in what person factors were available to use for the DIF analysis. However, we examined sex, age, affected hand details, treatment type, pain catastrophizing, Illness Perception overall score and the type of work performed, which is a more comprehensive evaluation of potential sources of differential item functioning than many Rasch papers. Lastly, although this analysis identified issues with the MHQ and attempts were made to correct them, recommendations cannot be made for improving the questionnaire because the issues with misfit, DIF, local dependence, and lack of unidimensionality could not be repaired.

## Conclusion

Although this study attempted to fit the brief MHQ to the Rasch model, issues with misfit, DIF, multidimensionality and local dependence could not be corrected successfully. Instead, the findings of this study can inform other researchers and clinicians on the strengths and shortcomings of the brief MHQ, and how these identified issues affect the use of the brief MHQ in clinical practice and research. The lack of unidimensionality suggests that the items represent more than one construct that are not sufficiently statistically related to be used for estimating relationships representing a singular construct of hand disability, and that it is not valid to sum the scores of the brief MHQ. Clinicians and researchers should consider the potential for inaccuracies if the total score is used for comparisons. Clinicians and researchers should also consider the item biases that exist for different demographic variables across several items. True between-group differences may be masked by item bias. Based on these findings, the 37-item MHQ may be a more suitable measure of hand disability for patients with thumb OA. Future Rasch analyses are required in other samples in an attempt to improve the fit of the brief MHQ to the Rasch model and determine if the concerns raised by this analysis are also true.

## Data Availability

The data that support the findings of this study are available from The Hand-Wrist Study group, but restrictions apply to the availability of these data, which were used under license for the current study, and so are not publicly available. Data are however available from the authors upon reasonable request and with permission of The Hand-Wrist Study group. The original data was obtained from: Selles RW, Wouters RM, Poelstra R, van der Oest MJ, Porsius JT, Hovius SE, Moojen TM, van Kooij Y, Pennehouat PY, van Huis R, Vermeulen GM. Routine health outcome measurement: Development, design, and implementation of the Hand and Wrist Cohort. Plastic and reconstructive surgery. 2020 Apr 30;146(2):343–54.

## Abbreviations

ANOVA: Analysis of variance
brief MHQ: Brief Michigan Hand Questionnaire
DIF: Differential item functioning
IPS: Illness Perception overall score
MHQ: Michigan Hand Questionnaire
OA: Osteoarthritis
PCS: Pain Catastrophizing Scale
PSI: Person Separation Index
R: Correlation
SD: Standard deviation
χ^2^: Chi Squared
